# Sex Differences in Age-Related Decline of Urinary Insulin-Like Growth Factor-Binding Protein-3 Levels in Adult Bonobos and Chimpanzees

**DOI:** 10.3389/fendo.2016.00118

**Published:** 2016-08-23

**Authors:** Verena Behringer, Stefan A. Wudy, Werner F. Blum, Jeroen M. G. Stevens, Thomas Remer, Christophe Boesch, Gottfried Hohmann

**Affiliations:** ^1^Department of Primatology, Max Planck Institute for Evolutionary Anthropology, Leipzig, Germany; ^2^Laboratory for Translational Hormone Analytics in Paediatric Endocrinology, Center of Child and Adolescent Medicine, Justus-Liebig-University, Giessen, Germany; ^3^Centre for Research and Conservation, Royal Zoological Society of Antwerp, Antwerp, Belgium; ^4^DONALD Study Dortmund, IEL-Nutritional Epidemiology, University of Bonn, Dortmund, Germany

**Keywords:** aging pattern, urinary marker, IGFBP-3, primate, ape species, *pan paniscus*, *pan troglodytes*

## Abstract

There is increasing interest in the characterization of normative senescence in humans. To assess to what extent aging patterns in humans are unique, comparative data from closely related species, such as non-human primates, can be very useful. Here, we use data from bonobos and chimpanzees, two closely related species that share a common ancestor with humans, to explore physiological markers that are indicative of aging processes. Many studies on aging in humans focus on the somatotropic axis, consisting of growth hormone (GH), insulin-like growth factors (IGFs), and IGF binding proteins (IGFBPs). In humans, IGFBP-3 levels decline steadily with increasing age. We used urinary IGFBP-3 levels as an alternative endocrine marker for IGF-I to identify the temporal pattern known to be related with age-related changes in cell proliferation, growth, and apoptosis. We measured urinary IGFBP-3 levels in samples from 71 bonobos and 102 chimpanzees. Focusing on samples from individuals aged 10 years or older, we found that urinary IGFBP-3 levels decline in both ape species with increasing age. However, in both species, females start with higher urinary IGFBP-3 levels than males, experience a steeper decline with increasing age, and converge with male levels around the age of 30–35 years. Our measurements of urinary IGFBP-3 levels indicate that bonobos and chimpanzees mirror human patterns of age-related decline in IGFBP-3 in older individuals (<10 years) of both sexes. Moreover, such as humans, both ape species show sex-specific differences in IGFBP-3 levels with females having higher levels than males, a result that correlates with sex differences in life expectancy. Using changes in urinary IGFBP-3 levels as a proxy for changes in GH and IGF-I levels that mark age-related changes in cell proliferation, this approach provides an opportunity to investigate trade-offs in life-history strategies in cross-sectional and in longitudinal studies, both in captivity and in the wild.

## Introduction

Compared to other non-human primates, human life span is longer and longevity increases progressively in all age classes, which translates into an overall “aging” of the human population. There is an increasing interest in understanding the biological phenomenon of advanced life stages, not only in disease etiology but also in characterizing normative senescence (1, 2). Senescence describes the time from full maturity to death and is characterized by a decline of the integrity and function of organic systems, decreased reproduction, and increased mortality (2, 3). Ethical and practical issues constrain investigations of such topics in humans to some degree, and data from non-human primates are considered to offer a good alternative model.

Non-human primates have relatively long life expectancies and in most species, individuals experience a phase of life that is characterized by visible signs of senescence (4). Additionally, the time period during which apparently healthy individuals develop alterations in behavior and age-related dysfunctions is longer in non-human primates compared to other animals (2). These two factors make non-human primates particularly interesting for exploring phenotypic traits of human senescence in an evolutionary context.

Like humans, many non-human primate species show consistent sex differences in aging, with adult males having shorter life spans and higher age-specific mortality than adult females (5–7). In captive and wild chimpanzees, females have on average a longer life span than males [e.g., Ref. (8–10)]. In wild bonobos, life expectancy and maximum life span are not yet well known, but life expectancy in captivity tends to be longer in females than in males (8, 11). The reasons for the observed sex differences in life expectancy remain to be explored. However, evidence from studies suggests that sex differences in life expectancy may be due to health status and/or behavioral strategies (9, 12, 13). Another possible explanation is that the sexes may differ in terms of age-related variation of physiological constitution. The goal of our study is to establish an endocrine marker to investigate aging processes in bonobos and chimpanzees.

Many studies on senescence in humans focused on the somatotropic axis, a multicomponent network consisting of growth hormone (GH), insulin-like growth factors (IGFs), IGF binding proteins (IGFBPs), and IGF receptors and IGFBP proteases *(BP-*Pr*)*. The components of the somatotropic axis are involved in the regulation of metabolism and somatic growth, as well as proliferation, survival, differentiation, and motility of cells by activating multiple signaling pathways [e.g., Ref. (14–16)].

Growth hormone stimulates cell proliferation and regeneration and is a major endocrine regulator of postnatal growth in mammals (17). Secretion of GH is regulated by negative feedback of serum glucose, free fatty acids, and IGF-I (18, 19).

IGF binding proteins are part of the IGF family of growth factors [e.g., Ref. (20, 21)]. In humans and non-human primates, IGFs exist in two forms, IGF-I and IGF-II (15). Both are synthesized mainly in the liver, and IGF-I increases in response to GH [e.g., Ref. (22, 23)]. In turn, rising IGF-I levels feedback on the hypothalamus and pituitary and inhibit GH release (24–26). The secretion of IGF-I is GH dependent (25–27) and, therefore, can serve as a surrogate marker for anabolic GH activity.

*In vitro* studies and animal experiments showed synergistic actions of GH and IGF-I on carbohydrate and lipid metabolism [e.g., Ref. (25, 28, 29)]. In addition, IGF-I is antiapoptotic and stimulates cell mitogenesis, survival, differentiation, and somatic growth [e.g., Ref. (14, 15, 23)]. Furthermore, IGF-I acts synergistically with insulin in the postprandial period as a hypoglycemic hormone (15). The amount of circulating IGF-I is determined by its production rate and metabolic clearance, and is modulated by IGFBPs that limit the availability of IGF to the IGF receptor (23). About 1% of blood IGF-I circulates in its free, unbound form, whereas most circulating IGF-I (about 99%) is bound to IGFBPs (30).

In humans, six IGFBPs have been identified and constitute an elaborate transport and regulatory system for IGF-I, and IGF-I-independent actions [e.g., Ref. (31)]. The main carrier for IGF in serum is IGFBP-3. It constitutes over 95% of the total IGFBP on a molar basis in humans (16, 27). IGFBP-3 is synthesized mainly in the liver (16, 28, 32). Approximately 80% of IGF-I is bound to IGFBP-3, which then forms a large molecular weight complex with an acid-labile subunit (ALS) [e.g., Ref. (17, 33)]. While the half-life of unbound IGFBP-3 is between 30 and 90 min and that of free IGF-I is less than 10 min, the half-life of the complex is about 12 h (15). By extending the serum half-life of IGF-I, IGFBP-3 affects cellular growth and the availability of IGF to receptors (23, 25, 34). Besides these functions, the complex may serve as an intravascular reservoir of IGFs that has the potential to strengthen or inhibit the activity of IGFs [e.g., Ref. (28, 34–36)]. In addition to the modulatory effect on IGF, IGFBP-3 induces cell apoptosis and inhibition of proliferation [e.g., Ref. (14, 23, 37)].

In humans, the somatotropic axis is age dependent (22). Serum IGF-I levels are low in infancy and childhood, then peak during adolescence, and decrease gradually with increasing age [e.g., Ref. (16, 36, 38, 39)]. IGFBP-3 levels undergo similar changes: values are low at birth, increase rapidly during the first weeks of life, and more slowly during later stages until they reach their maxima during puberty. From 18 years onward circulating IGFBP-3 levels decline steadily [e.g., Ref. (15, 33, 36, 40)]. In adults of both sexes, blood IGF-I levels decrease with age (24), and thereby contribute to age-related pathogenesis (41). Apart from age-related changes, various studies have reported sex-specific differences in IGF-I and IGFBP-3 levels: while average IGF-I and IGFBP-3 levels in elderly humans did not differ (20), IGFBP-3 levels were significantly higher in girls than in boys (38).

From neonates to the elderly, the components of the somatotropic axis are critical for survival [e.g., Ref. (42)]. A reduction in GH secretion in combination with reduced production of IGF-I contributes to an increase in fat mass and a decrease in muscle tissue (43). A reduction in IGF-I activity is associated with morbidity, increasing risk to develop cardiovascular disease, osteoporosis, and diabetes (29). Other studies have proposed a link between high IGF-I levels and the risk of cancer [e.g., Ref. (44)]. The correlation of age-related changes of IGF-I and IGFBP-3 levels may be due to a decline in GH secretion (43), indicating a causal relationship of the activity of the three components (22).

The age-related changes associated with an increase of IGF-I levels during puberty in humans have also been found in non-human primates, such as macaques (45–47), baboons (48–50), mandrills (51), gibbons (52), and chimpanzees (53). As in humans, IGFBP-3 values of rhesus macaques and baboons reached their maxima during puberty [e.g., Ref. (45, 48, 54)]. Examining IGF-I levels in adult macaques and baboons showed that in both species, serum IGF-I levels decreased with age (55, 56), which is comparable with the human aging pattern.

In non-human primates, adult serum IGF-I levels of males were 30% higher than in females in baboons (55), but other studies did not detect such differences in baboons and mangabeys (54, 57). IGF-I levels are higher in young female chimpanzees than in males (53), whereas in baboons and mandrills the opposite was shown (49, 51).

Most studies investigating physiological aging patterns in chimpanzees (58, 59) focused on topics, such as reproductive senescence (60) and age-related changes of neural structures [e.g., Ref. (61, 62)]. Moreover, to our knowledge, no study has explored senescence in bonobos, the sister species of chimpanzees. Therefore, the aim of the present study is to use measurements of urinary IGFBP-3 levels from captive adult bonobos and chimpanzees of both sexes to address the following questions: (1) Do urinary IGFBP-3 levels of adult chimpanzees and bonobos gradually decline with age? (2) Do females and males in either species differ in their overall levels of urinary IGFBP-3? (3) Do age-related changes in urinary IGFBP-3 levels of bonobos and chimpanzees reflect the observed sex difference in life expectancy?

When exploring the impact of the somatotropic axis on development, sex, and aging in non-human primates studies used blood samples. Given the ethical constraints imposed by guidelines of animal welfare, there is a need for alternative approaches, based on sample matrices that can be collected non-invasively. Although the IGFBP-3 concentration is approximately three times lower in urine compared to serum (35, 37, 63), urine IGFBP-3 measures can be used as a physiological marker to assess GH-IGFs aging patterns in hominoids and other primates. Urinary IGFBP-3 has been measured in urine of healthy humans with Western ligand blots (35, 64) and with immunoassay [e.g., Ref. (63, 65)]. IGFBP-3 levels reflect the GH secretory state, they are significantly correlated with serum IGF-I levels (27, 45, 66). Using physiological markers that can be extracted from non-invasively collected samples has several advantages. First, they facilitate monitoring age-related changes over time, collection of longitudinal records, construction of life-history profiles, and assessment of fitness (57, 67). Second, the technique can be applied to individuals living in natural environments.

## Materials and Methods

We measured IGFBP-3 levels in urine samples collected at random from 71 bonobos (31 males, 40 females) and 102 chimpanzees (33 males, 69 females). All samples were collected non-invasively from apes housed in zoo facilities (Table S1 in Supplementary Material) in accordance with the recommendations of the NIH published standards. Details on the sampling protocol as well as the transport and storage of samples have been published previously (68). In brief, urine samples were collected directly from the urine stream or taken off the ground, only when the individual could be identified and when contamination with feces could be excluded. After, the samples were immediately frozen in the zoo, and transported frozen to the Max Planck Institute for Evolutionary Anthropology in Leipzig, Germany. In order to explore changes in IGFBP-3 levels during advanced life stages, we focused our analyses on individuals of 10 years or older (bonobo age range: 10–55 years, average: 23 years; chimpanzee age range: 10–49 years, average: 23 years). For 54 of the 71 captive bonobos the exact birthdate was available from the studbook. For the chimpanzees, exact birthdates were available for 81 individuals. For six chimpanzees, only the year and month were known, and for these we set the day of birth to the 15th of the respective month. For the remaining individuals, only the year of birth was known, and in these cases the day of birth was set to June 15th of the respective year (12 females and 5 males bonobos, 13 females and 2 males chimpanzees).

### Urinary IGFBP-3 Analyses

Urinary IGFBP-3 concentrations were measured by a radioimmunoassay (RIA) developed for human IGFBP-3 detection (40). For assay validation, urine samples from bonobos and chimpanzees were serially diluted, and dilutions were found to parallel the standard curve (Figure S1 in Supplementary Material). Moreover, urine samples from each species were spiked with 2.5, 5, and 10 ng/ml human IGFBP-3, respectively. On average, recovery was 111 ± 8%, ranging from 99.0% (2.5 ng/ml spike) to 122.9% (5 ng/ml spike). Intra-assay and inter-assay coefficients of variation were 1.9% and 9.2%, respectively. Based on these results, the RIA was considered to be appropriate for measuring IGFBP-3 concentrations in urine samples of bonobos and chimpanzees.

Urine samples were adjusted for their volume, because the concentration of hormones in urine varies with fluid intake and the hydration status (69). For chimpanzees, it is suggested to correct urine volume using specific gravity (SG) [e.g., Ref. (70)]. SG is the ratio of the density of a urine sample relative to the density of water. We measured SG with a digital handheld refractometer (TEC, Ober-Ramstadt, Germany) and calculated urinary IGFBP-3 (nanogram/milliliter) corrected for SG (69, 71). The SG population average in our study was 1.007.

### Statistical Analyses

A general linear mixed model [GLMM (72)] was used to explore the influence of species, sex, and chronological age on urinary IGFBP-3 levels in bonobos and chimpanzees. The model was run in R (73) using the function lmer provided in the package lme4 (74).

We used a GLMM to investigate the impact of the predictor variables, i.e., species, sex, age (chronologic age in years), and sampling time (predictors with fixed effects) on the log-transformed response variable: urinary IGFBP-3 levels. The model included these four main effects as well as a three-way interaction between species, sex, and age, and all two-way interactions between them. These interactions were included in the model, because we expected that the age-dependent changes of urinary IGFBP-3 levels may differ between bonobos and chimpanzees and/or between males and females of each species, and that the degree of sex differences could be species specific. As a random effect, we included location (the zoo housing a specific individual) to control for a possible influence of relevant animal husbandry conditions of zoos (e.g., diet and group size). Furthermore, we included random slopes of age at sampling time within zoo to keep type I error rates at the nominal level of 5% (75). Age was square root transformed. The transformed age as well as the time of sample collection (to control for diurnal variation) was *z*-transformed to a mean of zero and a SD of one to achieve comparable estimates (76).

For the model, the required normal distribution and homogeneity of residuals were assessed by visual inspections of a histogram, a q–q plot of the residuals, and by plotting residuals against fitted values. All model assumptions were met. Model stability was tested by excluding zoos one by one, which did not indicate any obvious influence of this random effect. To test for collinearity, we examined Variance Inflation Factors [VIF (77)] using the function vif of the R-package car (78) applied to a standard linear model excluding random effects. These indicated that collinearity was not an obvious issue (maximum VIF: 1.044).

Investigating the significance of the fixed effects species, sex, age, and all their interactions as a whole (79), we compared the full model with a null model, excluding the predictor variables and the interactions, but retaining time of sample collection, the random effect of zoo, as well as the random slopes component, using a likelihood ratio test [Ref. (80); R function “anova”]. The data were bootstrapped 1000 times to obtain parameter coefficients. Significance for all tests was set at the *P* = 0.05 level.

Data for reproductive status were only available for female bonobos. To test to what extend female reproductive status (cycling, lactating, and pregnant) affect urinary IGFBP-3 levels, we used a one-way ANOVA. To achieve normal distribution of the response variable, IGFBP-3 levels were log-transformed. This analysis was also conducted in R using the function anova.

## Results

**Figure 1:**
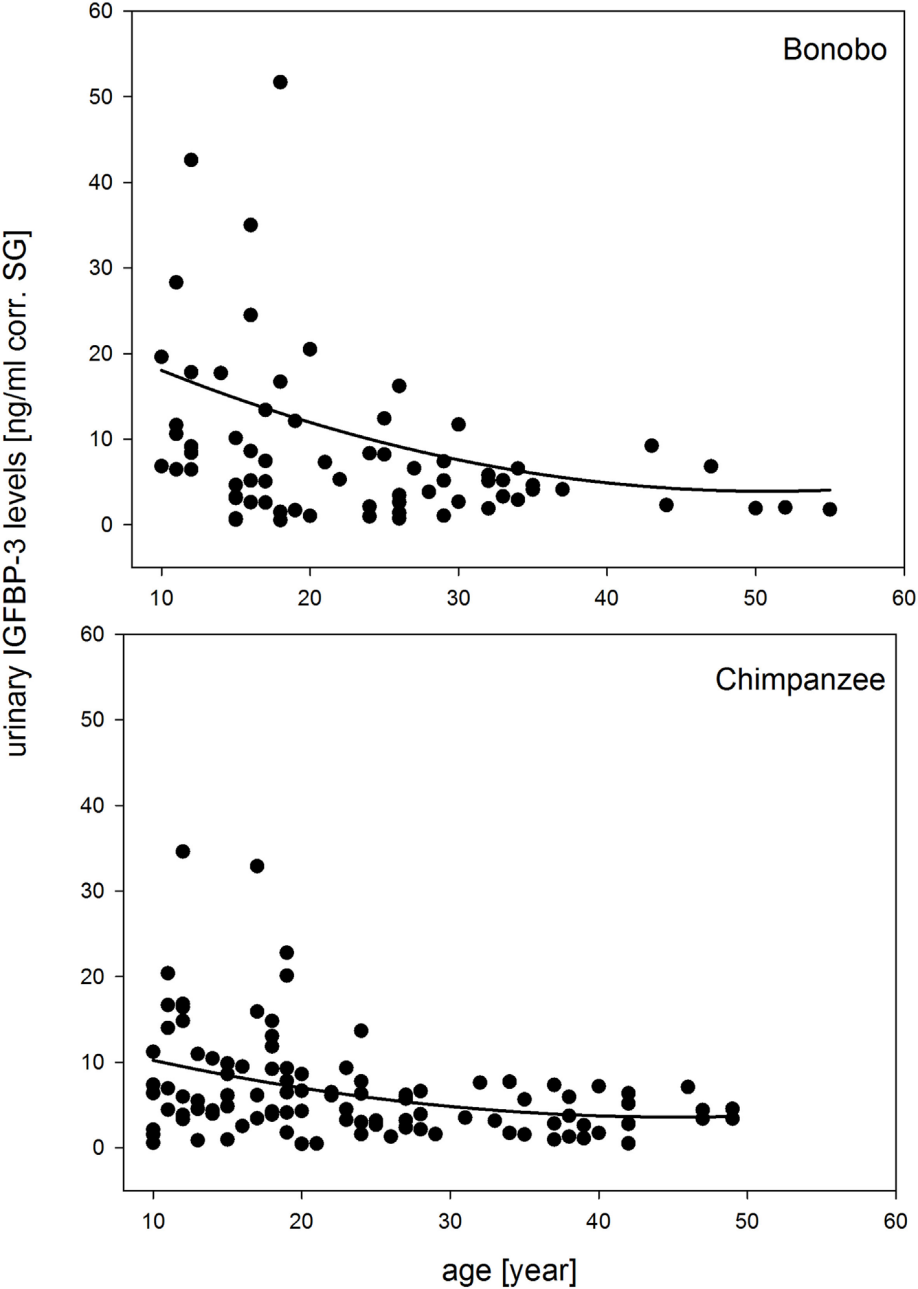
**Measures of urinary IGFBP-3 levels corrected for specific gravity (corr. SG) from bonobos (upper plot) and chimpanzees (lower plot) in relation to chronologic age**. Sample sizes: *N*_bonobos_ = 71 (31 males, 40 females), *N*_chimpanzees_ = 102 (33 males, 69 females).

The full-null model comparison revealed that sex, species, and chronologic age had significant effects on urinary IGFBP-3 levels (χ^2^ = 35.298, df = 7, *P* < 0.001). The three-way interaction of sex, species, and chronologic age was not significant (estimate = −0.165, SE = 0.235, *z*-value = −0.703, *P* = 0.485) and was, therefore, excluded from the model. In the reduced model, all two-way interactions were included. The interaction of species with age was not significant, because urinary IGFBP-3 levels in both species showed a similar decrease with age (Figure 1).

**Figure 2:**
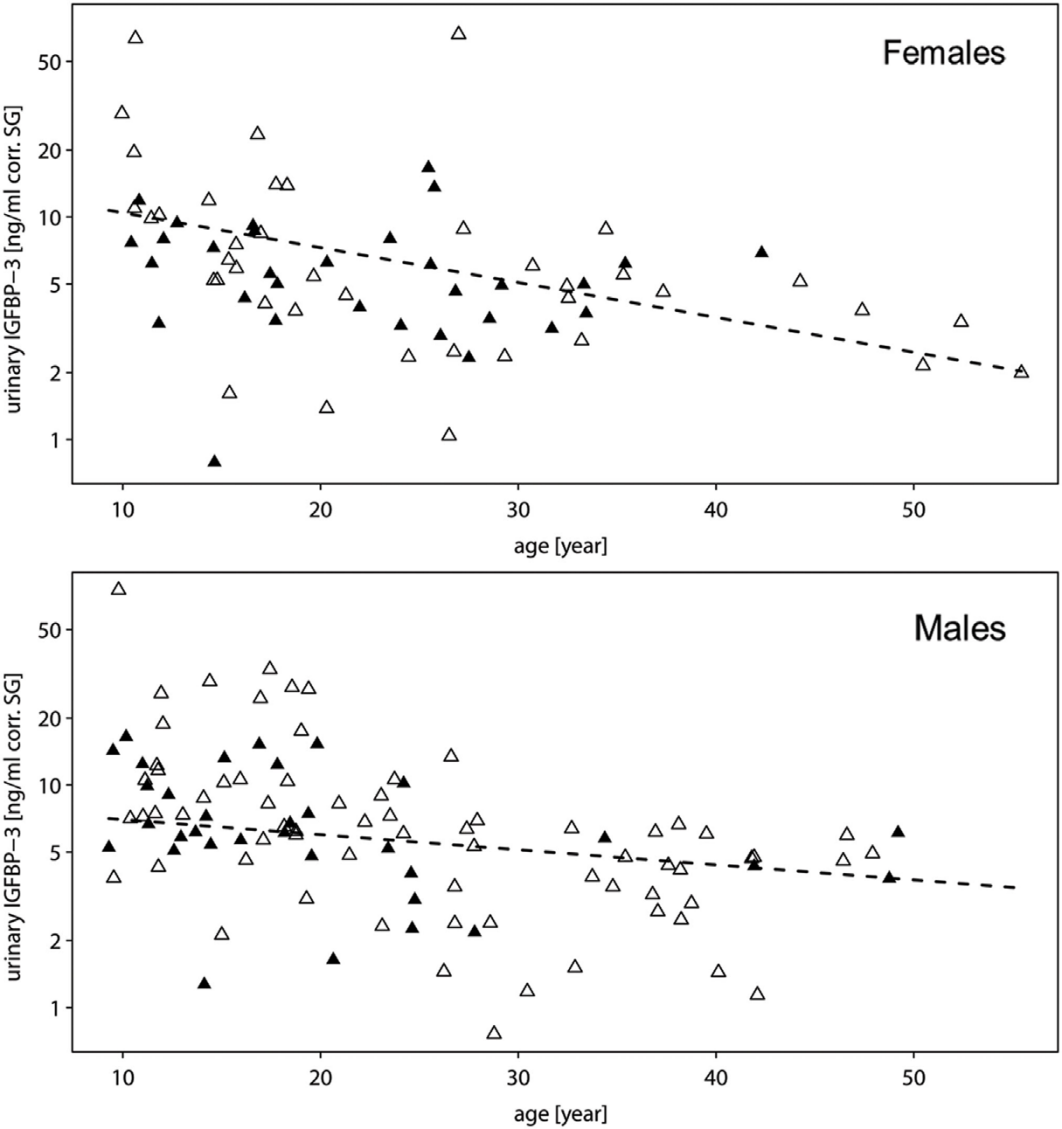
**Measures of urinary IGFBP-3 levels corrected for specific gravity (corr. SG) from female (upper plot) and male (lower plot) bonobos (open triangles) and chimpanzees (filled triangles) in relation to chronologic age**. The *y*-axis is displayed on a log scale. Sample sizes: *N*_bonobos_ = 71 (31 males, 40 females), *N*_chimpanzees_ = 102 (33 males, 69 females).

All two-way interactions, including species were removed from the model. The interaction of chronologic age with sex showed a trend (Table 1A). Therefore, we ran a second reduced model with the interaction of chronologic age and sex, and species as predictor. The interaction of chronologic age with sex remained a trend (Table 1B), demonstrating that age-related changes in urinary IGFBP-3 levels differ between the sexes (Figure 2).

**Table 1:** **Results of the two reduced general linear mixed models obtained by analyzing urinary IGFBP-3 levels from chimpanzees and bonobos of both sexes: (A) with species, chronologic age, and sex in interactions; and (B) with the interaction of sex and chronologic age, and species as fixed effect**.

Term	Estimate	SE	DF	χ^2^	*P*-value
**(A) With species, chronologic age, and sex in interactions**					
Intercept	1.826	0.129			
Time of sample collection	−0.040	0.056	1	0.501	0.479
Chronologic age*sex	0.224	0.115	1	3.732	**0.053**
Chronologic age*species	−0.068	0.107	1	0.394	0.530
Sex*species	0.052	0.222	1	0.056	0.813
**(B) With the interaction of sex and chronologic age, and species as fixed effect**					
Intercept	1.8211	0.119			
Time of sample collection	−0.038	0.056	1	0.457	0.499
Species	0.103	0.134	1	0.582	0.466
Chronologic age*sex	0.223	0.115	1	3.691	**0.055**

In both models, time of sample collection is a fixed effect and zoo facility is a random effect (*refers to an interaction term and bold numbers indicate significance).

**Figure 3:**
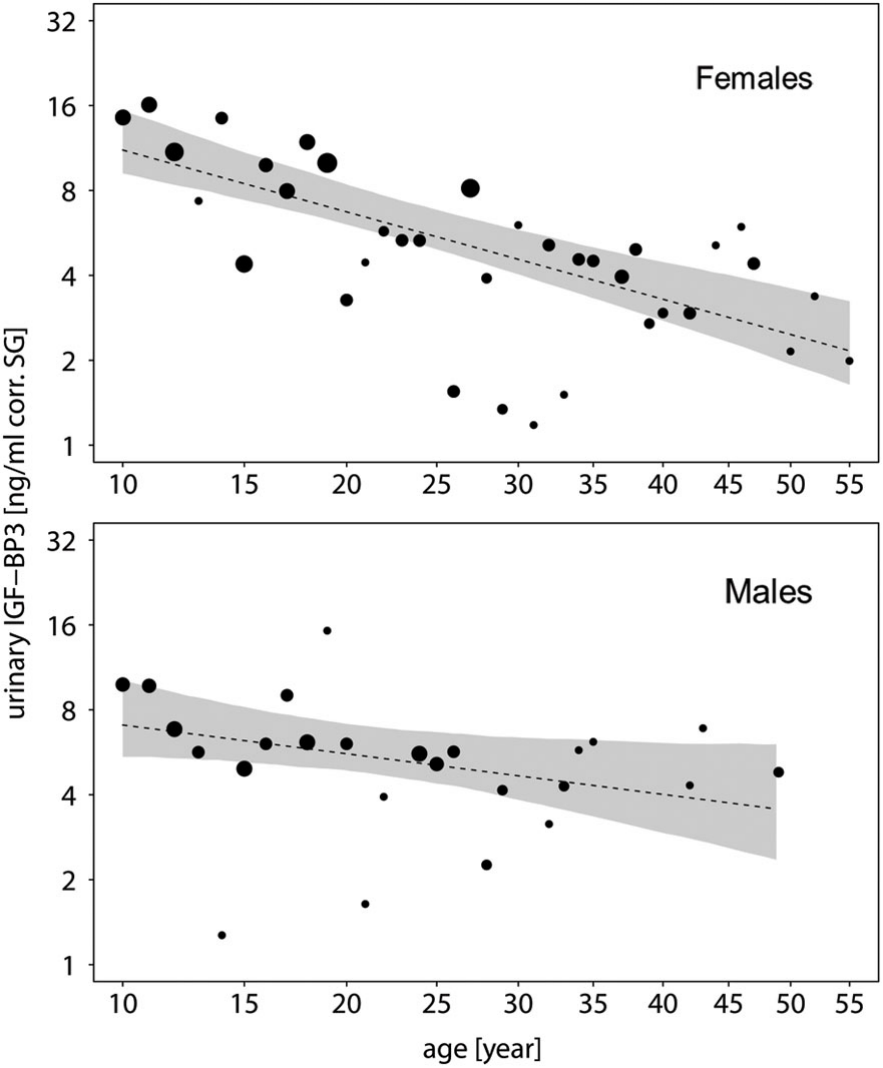
**Measures of urinary IGFBP-3 levels corrected for specific gravity (corr. SG) obtained from female (upper plot) and male (lower plot) bonobos and chimpanzees in relation to their chronologic age (year)**. Shaded areas represent bootstrapped 95% confidence intervals for expected urinary IGFBP-3 levels. The area of the circles corresponds to the number of individuals. The *y*-axis is displayed on a log scale. Sample sizes: *N*_bonobos_ = 71 (31 males, 40 females), *N*_chimpanzees_ = 102 (33 males, 69 females).

To further explore this, we built subsets for each sex and ran a second reduced model for each subset without the main effect of sex. In both sexes, urinary IGFBP-3 levels decreased significantly with chronologic age, but the decreasing slope was steeper in females when compared with males (males: estimate = −0.200, SE = 0.082, *z*-value = −2.433, *P* = 0.018; females: estimate = −0.422, SE = 0.068, *z*-value = −6.157, *P* = <0.001) (Figure 3). Moreover, urinary IGFBP-3 levels from females started from a higher level than samples from males and converged with male levels around the age of 30 (Figure 3).

According to this analysis, reproductive status of female bonobos did not affect urinary IGFBP-3 levels (*F*_2,37_ = 0.219, *P* = 0.804).

## Discussion

Little is known about the process of senescence in chimpanzees and to our knowledge nothing has been published about the topic for bonobos. However, there is consensus that information on aging and senescence from our closest living relatives offers a model to better understand the biological components of aging and senescence in our own species [e.g., Ref. (1, 5, 81)]. In this study, we used urinary IGFBP-3 levels as an alternative endocrine marker for IGF-I to identify the temporal pattern known to be related with age-related changes in cell proliferation, growth, and apoptosis. Focusing on samples from individuals aged 10 years or older, we found the following: urinary IGFBP-3 levels decline with increasing age. In both species, females start with higher urinary IGFBP-3 levels than males, experience a steeper decline with increasing age, and converge with male levels around the age of 30 years.

The age-related decrease in urinary IGFBP-3 levels obtained in our study from chimpanzees and bonobos correspond with a significant decline of serum and urinary IGFBP-3 levels with increasing age (after mid-puberty) in humans (36, 64, 82, 83). Given that a decrease with age in serum IGF-I and IGFBP-3 levels is consistent with an age-related decline in GH secretion in humans (43), we assume that the gradual decline in urinary IGFBP-3 levels in the two ape species indicates a corresponding change of GH and IGF-I levels. The reduced secretion of GH and IGF-I corresponds to the somatopause of humans (20, 84), an important determinant in age-related changes in body composition and function (24, 39, 85).

Our measures of urinary IGFBP-3 levels indicate that bonobos and chimpanzees experience an aging pattern in this biomarker, similar to that of modern humans, and sex-specific differences in IGFBP-3 levels are another trait that the three species share. At 10 years of age, female bonobos and chimpanzees have higher levels of urinary IFGBP-3 and converge with males at 30 years of age. The same sex difference has been reported from several human studies, where average serum IGFBP-3 levels were significantly higher in women than in men [e.g., Ref. (36, 82, 83, 86, 87)]. Data for a quantitative comparison between humans, bonobos, and chimpanzees are not yet available. First, most data from humans are based on serum samples, while our data are from urine samples. Second, species-differences in life time expectancy complicate direct comparison of hormone levels. Third, laboratory methods for measuring IGFBP-3 levels vary across studies.

Previous studies have suggested that the decrease in IGFBP-3/IGF-I levels may indicate progressive senescence in hominids (29). As studies of humans have shown, our study of bonobos and chimpanzees found that the sex-specific decline of IFGBP-3 levels correlates with sex differences in life expectancy. In western human societies, women live on average 5–7 years longer (10% more) than men (2). In captive apes, females accounted for the majority of individuals older than 40 years. This indicates a greater survivorship in females than males, and resembles the female-biased life expectancy in modern humans (88, 89).

The significantly higher IGFBP-3 levels in elderly women correspond with lower bioavailable IGF-I levels, whereas in men, the opposite is the case (82, 83, 86, 87). Therefore, one can speculate that the higher IGFBP-3 levels found in the females of our study reflect lower IGF-I levels. High IGF-I levels may promote the functioning of individual tissues and organs, but have negative pleiotropic effects on longevity as IGF-I is associated with increased oxidative damage and risk of pathology [e.g., Ref. (41, 90)]. In chimpanzees, kidney and liver function decreases significantly with older age. These changes become evident in males around 25–30 years and in females between 30 and 35 years (59). IGFBP-3 is produced by sinusoidal cells (22), and an earlier decrease in liver function in males could be possibly related to lower IGFBP-3 levels.

Another explanation for the sex differences in IGFBP-3 levels could be the relationship between IGFBP-3 and reproduction. First, pregnancy and breastfeeding are associated with increased IGFBP-3 levels in humans (83, 91, 92) and IGFs are synthesized by the placenta during pregnancy (93). However, in our study, 50% of the female bonobos (20 individuals) were lactating and 17.5% (seven individuals) were pregnant, but their urinary IGFBP-3 levels did not differ in comparison to cycling females. Unfortunately, the reproductive status of the female chimpanzees in our study was not sufficiently documented, thereby prohibiting us to include this parameter as a confounding factor. Second, IGFs regulate gametogenesis and reproductive function (48). In humans and in captive and wild chimpanzees, birth rates decline at the beginning of the fourth decade of life (94). In women, reproductive senescence is characterized by menopause – a sudden decline in fecundity around 50 years of age [e.g., Ref. (81)]. Although female chimpanzees and bonobos show a similar decline in fecundity at older ages, it is unclear if they experience menopause. However, demographic data from wild and captive chimpanzees indicate that females do experience a post-reproductive period [e.g., Ref. (12, 94, 95)]. In our study, only five female bonobos (12.5%) and only eight female chimpanzees (11.6%) were older than 40 years.

One limitation of our study is its cross-sectional nature, which cannot control for the possible effects of inter-individual variation in development (28). Another limitation is that interventions of management and husbandry are likely to influence hormone levels. However, the advantage of research on captive apes is the exact knowledge of age of the zoo-born individuals, the majority of apes, and their long life expectancy (1, 4).

Growth requires input from both, the thyroid axis and the somatotropic axis (96). Thyroid hormones may directly regulate circulating IGFBP-3 levels (97, 98). Overall, the age-related decline in urinary IGFBP-3 found in this study corresponds with urinary total triiodothyronine (TT3) changes reported from bonobos and chimpanzees (68). However, the sex difference in urinary IGFBP-3 levels observed in both species was not found in urinary TT3 levels from chimpanzees. By contrast, bonobos show sex differences in both markers, but in opposite directions, with females having significantly higher urinary IGFBP-3 and lower TT3 levels than males. The implication of the different levels in these hormones in males and females on health and longevity could be explored in future long-term data sets.

Because IGFBP-3 levels can be measured in urine samples, this biomarker can be easily used for larger numbers of individuals, thereby overcoming limitations of small sample sizes that are characteristic of invasive sampling techniques (57, 99). Moreover, it facilitates collection of repeated measures within individuals to monitor more accurately the process of senescence, which helps to understand the evolutionary theory of it. A central concept in the evolutionary theory of senescence is the idea that aging results from the investment in different life-history trade-offs (100). Therefore, measuring urinary IGFBP-3 levels as a proxy for changes in GH and IGF-I levels and, thus, as a gross estimate of cell proliferation and aging processes, provides an opportunity to investigate trade-offs in life-history strategies in cross-sectional as well as in longitudinal studies both in captivity and in the wild (90).

## Conclusion

Using a cross-sectional study design, we found that urinary IGFBP-3 levels decline with age in adult individuals of two closely related ape species, bonobos and chimpanzees. In both species, urinary IGFBP-3 levels of females started at a higher level and had a steeper age-related decline than in males. The age-related and sex-specific changes in urinary IGFBP-3 levels in bonobos and chimpanzees are similar to the patterns observed in modern humans. Moreover, in all three species, the sex-specific decline of IFGBP-3 levels is correlated with sex differences in lifetime expectancy, but in bonobos and chimpanzees, the causal link between lifetime expectancy and IGFBP-3 needs to be explored. Urinary IGFBP-3 levels can be used as an alternative endocrine marker for IGF-I to identify the temporal pattern known to be related with age-related changes in cell proliferation, growth, and apoptosis in bonobos and chimpanzees.

## Author Contributions

VB, TR, SW, JS, CB, and GH were involved in the conception and design of the study. VB was responsible for sample collection. WB and SW developed the assay and measured IGFBP-3. VB and GH analyzed the data. All authors were involved in interpretation of data and writing or editing of this manuscript. All authors approved the final version of the manuscript.

## Conflict of Interest Statement

No authors (or their institutions) of this manuscript received payment or services from a third party for any aspect of this manuscript. The research was conducted in the absence of any commercial or financial relationships that could be construed as a potential conflict of interest. We do not have patents or copyrights to declare.
