# Supplementary material for: Sex Differences in Age-Related Decline of Urinary Insulin-Like Growth Factor-Binding Protein-3 Levels in Adult Bonobos and Chimpanzees
Source: Front Endocrinol (Lausanne). 2016 Aug 23;7:118. doi: 10.3389/fendo.2016.00118 (PMC4994059; doi:10.3389/fendo.2016.00118)
Supplement: Supplementary file 1 [file Table_1.PDF]

Table S1: Participating zoos, countries, and number of specimens for each sex.

Species	Zoo	Country	Male	Female
Bonobo	Apenheul	Netherlands	1	3
Bonobo	Berlin	Germany	1	1
Bonobo	Cologne	Germany	2	2
Bonobo	Frankfurt	Germany	2	6
Bonobo	Leipzig	Germany	3	2
Bonobo	Milwaukee	USA	6	6
Bonobo	Planckendael	Belgium	6	4
Bonobo	Romagne	France	1	4
Bonobo	San Diego WAP*	USA	2	2
Bonobo	San Diego Zoo	USA	2	3
Bonobo	Stuttgart	Germany	1	6
Bonobo	Wuppertal	Germany	4	1
Chimpanzee	Aalborg	Denmark	1	1
Chimpanzee	Amsterdam	Netherlands	2	6
Chimpanzee	Arnhem	Netherlands	1	1
Chimpanzee	Augsburg	Germany	2	1
Chimpanzee	Belfast	UK	1	3
Chimpanzee	Boras	Sweden	-	1
Chimpanzee	Bratislava	Slovakia	2	-
Chimpanzee	Bremerhaven	Germany	1	-
Chimpanzee	Copenhagen	Denmark	2	-
Chimpanzee	Dudley	UK	-	2
Chimpanzee	Furuviksparken	Sweden	1	2
Chimpanzee	Gdansk	Poland	-	2
Chimpanzee	Gelsenkirchen	Germany	-	2
Chimpanzee	Givskud	Denmark	-	4
Chimpanzee	Halle	Germany	-	1
Chimpanzee	Heidelberg	Germany	1	4
Chimpanzee	Kristiansand	Norway	2	1
Chimpanzee	Leipzig	Germany	5	15
Chimpanzee	Ljubljana	Slovenia	2	2
Chimpanzee	Madrid	Spain	2	-
Chimpanzee	Malaga	Spain	1	4
Chimpanzee	Muenster	Germany	1	-
Chimpanzee	Munich	Germany	1	3
Chimpanzee	Ramatgan	Israel	1	3
Chimpanzee	St Andre	Portugal	1	1
Chimpanzee	Valencia	Spain	-	2
Chimpanzee	Verona	Italy	1	1

Chimpanzee	Veszprem	Hungary	1	2
Chimpanzee	Warsaw	Poland	1	5

* WAP = Wild animal park
