# Supplementary material for: Sex Differences in Age-Related Decline of Urinary Insulin-Like Growth Factor-Binding Protein-3 Levels in Adult Bonobos and Chimpanzees
Source: Front Endocrinol (Lausanne). 2016 Aug 23;7:118. doi: 10.3389/fendo.2016.00118 (PMC4994059; doi:10.3389/fendo.2016.00118)

Figure S1. Displacement of hIGFBP-3 tracer binding in the hIGFBP-3 radioimmunoassay by geometric dilutions of human and primate urine: hIGFBP-3 standard (solid circles), human urine (open circles), primate urine (solid squares). Serially diluted urine samples parallel the standard curve, confirming the validity of IGFBP-3 measurement in primate urine by this heterogeneous IGFBP-3 radioimmunoassay.

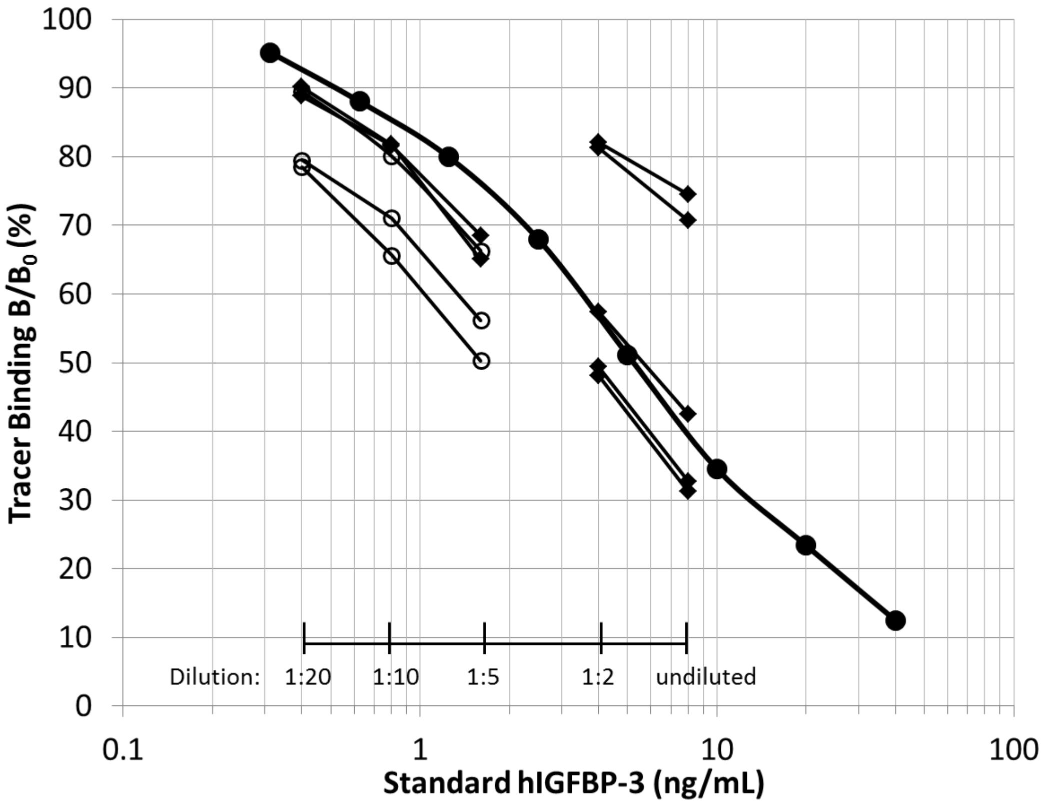

Supplement: Supplementary file 2 [file Image_1.PDF]
